# Lung cancer and concurrent or sequential lymphoma: Two case reports with hypersensitivity to bevacizumab and a review of the literature

**DOI:** 10.3892/ol.2014.2717

**Published:** 2014-11-20

**Authors:** ALDO PEZZUTO, ALESSIO PIRAINO, SALVATORE MARIOTTA

**Affiliations:** Cardiopulmonary Department, Sant’Andrea Hospital, Sapienza University, Rome 00189, Italy

**Keywords:** lung cancer, hypersensitivity to bevacizumab, sequential lymphoma, concurrent lymphoma

## Abstract

Non-small cell lung cancer (NSCLC) accounts for ~80% of all cases of lung cancer, and is the leading cause of cancer-related mortality worldwide. The majority of NSCLC cases of are diagnosed at an advanced stage. The outcome of patients with advanced NSCLC is poor with a median survival time of ~12 months in European and American populations. Lymphoproliferative disorders (LPDs) represent a heterogeneous group of expanding lymphoid cells, which occurs as a result of immune dysfunction. LPDs are often associated with primary solid cancers. We report two cases of LPD diagnosed concurrently and successively to NSCLC. The first case presents a 65-year-old female patient with advanced IV stage lung cancer, according to the International Association for the Study of Lung Cancer TNM staging system. The patient developed a concurrent lymphoma and was treated with first-line therapy including six cycles of gemcitabine and cisplatin, however, the patient experienced an adverse drug reaction to bevacizumab, which was administered after gemcitabine and prior to cisplatin. The second case presented a 74-year-old male patient diagnosed with large B cell lymphoma. The patient acheived remission of the illness, however, after one year the patient was diagnosed with squamous cell lung cancer. After three years, the patient underwent surgery, however disease recurrence was identified. Subsequently, the patient was treated with sterotactic radiotherapy and oral chemotherapy. A review of the associated literature was also conducted.

## Introduction

Non-small cell lung cancer (NSCLC) is the leading cause of cancer-related mortality, which accounts for 15% of all new cases of cancer globally and this type of cancer has been considered as nonimmunogenic (1). In Europe, lung cancer accounts for ~3.2 million new cancer cases, annually. The International Association for the Study of Lung Cancer TNM staging system identified four major stages of lung cancer, which correlate with significant differences in five-year survival rates (2). The five-year survival rate in patients with stage I cancer (70%) is significantly higher than that of patients with stage III A cancer (~25%)(3–5). Lymphoproliferative disorders (LPDs) are a heterogeneous group of biologically and clinically distinct neoplasms, including non-Hodgkin and Hodgkin’s lymphoma, which originate from the lymphoid organs. Previous studies have investigated the molecular pathogenesis of lymphoid malignancies (6,7). Interactions between the immune system and cancer development and progression have been previously demonstrated. Lung tumors can evoke a cellular antitumor immune response (8). High expression of CD8 and CD4 T cells and macrophages in tumor tissue has been demonstrated (9). A case of primary lung cancer complicated by lymphoma was described by Ohno *et al* (9). Additional cases of primary cancers associated with LPDs have been reported, including breast and non-small cell lung cancer, however, gastric cancer with large B cell lymphoma was found to occur most frequently (10–12). In this study, the possible interaction between the immune system and solid tumors was investigated. In addition, two cases of LPD diagnosed concurrently and successively to NSCLC are presented. The first case presents a patient with the concurrent diagnosis of lymphoma and lung cancer, who exhibited an hypersensitivity reaction to bevacizumab, and the second case presents a patient characterized by sequential diagnosis. Written informed consent was obtained from both patients.

## Case reports

### Case one

The first patient was a 65-year-old female with a smoking history of 40 pack years and a family history of blood hypertension. The patient had previously suffered from pneumonia one year prior to diagnosis and, clinically, the patient complained of mild dyspnoea and was admitted to the Cardiopulmonary Department of Sant’Andrea Hospital (Rome, Italy). Blood tests showed anemia with hemoglobin levels, 9.9 g/dl (normal range, 12.5–15 g/dl); platelet count, 360,000 μl (normal range, 150,000–450,000 μl; relative lymphocytosis (70%; normal range, ≤20%); creatinine levels, 1.3 mg/dl (normal range, 0.6–1 mg/dl); white blood cell count, 18.0×10^3^ μl (range, 4.3–10.8×10^3^ μl); and oncomarker carcinoembryonic antigen (CEA) levels, 7.5 ng/ml (normal range, 2.5–5 ng/ml).

**Figure 1:**
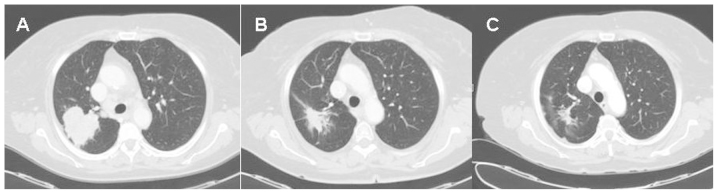
Case 1: (A) CT scan showing a lesion in the upper lobe of the right lung, (B) CT scan after three months of chemotherapy indicated a partial response and (C) CT scan showing complete response after six cycles of chemotherapy. CT, computed tomography.

A computed tomography (CT) scan was performed (Fig. 1A) and a right lung mass was discovered, located in the upper right lobe and 5 cm in diameter, with contrast enhancement. The mediastinal lymph nodes were found to be involved and thrombi were present in the right renal vein.

The patient subsequently underwent a transthoracic CT-guided needle biopsy. Immunohistochemistry revealed that the tissue was positive for thyroid transcription factor 1, CK67 and p63. Therefore, the patient was diagnosed with an adenosquamous tumor.

Lymphocyte typing and serum immunofixation tests performed on the patient’s blood sample showed, respectively, a high level of B cells 41% 1318 (up to 400) and B lymphocyte CD19^+^FMC^+^ presence, with an associated high intensity of light λ-chains. Therefore, the diagnosis was lymphoproliferative syndrome B, LNH type (Table I).

The patient was treated for lung cancer, with a first line therapy including six cycles of gemcitabine (1,000 mg/m^2^, days 1 and 8) and cisplatin (70 mg/m^2^, day 1) for 12 days, showing a good response (Fig. 1B). The patient had an adverse reaction to bevacizumab during the first cycle, which was adminstered after gemcitabine and prior to cisplatin, and for this reason it was not administered further. The symptoms of bevacizumab treatment were itching, urticaria, erythematous rash and facial swelling. Second line therapy was initiated with six cycles of vinorelbine (60 mg/m^2^, days 1 and 8) as a single agent for 12 days.

Blood samples after each course revealed a grade 4 neutropenia with a high level of lymphocytes (lymphocytes, 70%; neutrophils, 12%). The total white blood cell count was 4,800/μl. A CT scan performed after the second line therapy showed a partial response with lymph node down-staging (Fig. 1C). Following multidisciplinary evaluation by radiotherapists and hematologists, the case was considered amenable for local radiotherapy. Treatment of LPD, including chemotherapy and steroidal therapy, was delayed until the termination of this course of radiotherapy, and a follow-up strategy has been adopted until the time of writing, with a survival from diagnosis of ~23 months without objective signs of hematological disease.

### Case two

A 74-year-old male was referred to the Cardiopulmonary Department, Sant’Andrea Hospital, Sapienza University (Rome, Italy) for dyspnoea, weakness and chest pain. The patient was subsequently admitted to the Department of Lung Oncology (Sant’Andrea Hospital, Sapienza University). Analysis of the patient’s blood sample revealed lymphocytosis. Further investigation included a bone marrow biopsy, revealing an infiltration of large B-cell lymphoma CD20/79a^+^, stage IV (due to hepatic involvement) according to the European-American classification of lymphoid neoplasms (13).

The patient underwent chemotherapy with cyclophosphamide, prednisone, vincristine and doxorubicine. A positron emission tomography (PET)-CT scan following diagnosis revealed partial remission. Two months after diagnosis, due to persisting metabolic uptake shown by PET, a second-line therapy was started with cisplatin, dexhamethasone and rituximab. Subsequently, ibritumomab was used for the maintenance therapy and periodic follow-ups revealed the patient was in remission.

**Figure 2:**
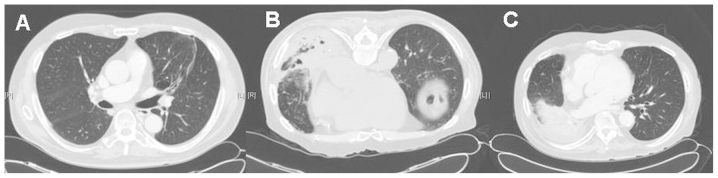
Case 2: (A) CT scan showing the lung after surgery, (B) CT scan three years after surgery showing recurrence of lung cancer and (C) CT scan after three cycles of chemotherapy revealed disease progression. CT, computed tomography.

The following year, the patient was diagnosed with squamous cell lung cancer (p63- and CEA-positive). The patient underwent a right superior lung lobectomy. The lung cancer stage was determined as stage Ib, according to the most recent staging system (14). A subsequent CT scan was performed after surgery (Fig. 2A), which revealed the results of the right superior lung lobectomy.

The patient acheived remission for three years, however, was then referred to the Cardiopulmonary Department, Sant’Andrea Hospital, Sapienza University for chest pain. Laboratory tests revealed leukocytosis with a high level of white blood cells (14,000 μl; normal range, 4,5000–10,000 μl) and an increased level of C-reactive protein (16 mg/dl; normal range, 0–0.5 mg/dl). Protein electrophoresis analysis revealed that γ globulin levels were also increased (normal range, 0.7–1.4 g/dl), and κ (normal range, 3.9–19.4 mg/l) and λ (normal range, 5.7–26.3 mg/ml) light chains were lower.

A chest CT scan revealed an irregular profile mass (Fig. 2B), which was confirmed as recurrent lung cancer after fine needle aspiration biopsy. A brain CT scan also revealed a metastatic lesion.

The patient underwent stereotactic radiotherapy over one day, 20 Gy of the total dose. Subsequently, six cycles of oral chemotherapy with 60 mg/m^2^ vinorelbine was initiated for 12 days. Restaging three months after the beginning of therapy showed a minimal response (Fig. 2C). The patient remained alive at the follow-up after three years, with pulmonary stable disease. Follow-up hematological and radiological CT scans were also planned.

## Discussion

It is well demonstrated that the population of cancer survivors has an increased risk of developing secondary cancers (8–16%). Leukemias and lymphomas generally arise in the first five years of treatment, whereas solid cancers such as breast, lung, gastrointestinal, brain, genitourinary cancers usually arise after five years (15–18). LPDs have three major categories: B-cell, NK/T-cell neoplasms and Hodgkin lymphoma (HL), according to the World Health Organization classification (19). Recently, a marked improvement in survival was achieved for patients with LPD, thus increasing the likelihood of secondary cancer (20,21). LPDs are the neoplasms most frequently involved in cases of multiple primary cancers (MPCs), as primary or subsequent (1).

The etiology of second primary cancers may be attributed to the interaction between the immune system and carcinogenetic factors; numerous risk factors have been described and identified as contributing to the development of LPD. These may act in association to result in a second malignancy, and include immunodeficiency states (22,23), viruses (particularly the Epstein-Barr virus), chemotherapeutic agents, pesticides and organic solvents, and exposure to ultraviolet radiation (23,24), A family history of LPD (24–28), but also genetic susceptibility (29), may be found. Shared risk factors, such as smoking and genetics, may cause certain malignancies to occur more frequently than expected. The study of multiple primary malignancies following LPD therefore has etiological and clinical importance. In a recent retrospective study on MPC by Demirci *et al* (30), the ability to diagnose a second cancer was shown to be earlier and more accurate than in past reports. In a population of 242 patients, the mean time for diagnosis of second malignancies was 55.5 months, whereas a third cancer was diagnosed after 22 months of the second neoplasm. In the same study, the incidence of solid tumors compared with LPD as a second cancer was equal, contrary to the results of previous studies (20,31,32). The frequencies of primary and secondary diagnoses of LPD were the same (27). NSCLC was the second most frequently diagnosed solid tumor after LPD, the first being breast cancer. Diffuse large B-cell lymphoma as a subsequent cancer was the most frequent subtype of non-HL (NHL). Markedly high incidences of lung cancer, melanoma, soft tissue sarcoma, NHL, acute myeloid leukemia, genitourinary cancers, and head and neck cancer have been reported in lymphoma survivors (1,30,33). In the present case, the patient in case two had a synchronous diagnosis of LDP and lung cancer. Tumors are considered synchronous when the period of time between the diagnoses of the primary and subsequent malignancies is less than or equal to six months (10).

A synchronous diagnosis of thoracic MPC with lymphoma and myeloma has previously been reported (10). BALT lymphoma has been found in patients affected by lung adenocarcinoma in six cases and by squamous carcinoma in one case (10,30,33–37). One case of lung adenocarcinoma and mantle cell lymphoma of the pleura, and one case of unspecified lung cancer associated with tracheal mucosa-associated lymphoid tissue lymphoma have also been reported. The most common type of primary pulmonary lymphoma is bronchus-associated lymphoid tissue in the lungs (38). The concurrent presence of two solid and hematic neoplasms may affect treatment choice, as solid tumors may cause a greater sensitivity to anticancer drugs and may lead to a worse prognosis. Numerous chemotherapeutic agents, including vincristine, carboplatin and cyclosporine are used as a single treatment, as well as in combination with other drugs. However, these drugs may induce hypersensitivity reactions.

Immediate hypersensitivity reactions for the majority of chemotherapeutic agents were described in a review by Visittsunthorn *et al* in the pediatric population (39). Symptoms were either moderate or mild, and early reaction onset is generally a result of IgE-mediated hypersensitivity. Delayed reactions could be explained by a release of TNF-α and interleukin 6 during treatment (40).

Vincristine as well as carboplatin, cychlophosphamide and cyclosporine may be used as monotherapies, as well as for combined treatments. Bevacizumab has not been yet presented in these settings, but the unusual manifestation we observed while using this drug in a ‘double-cancer’ patient could hide more complex mechanisms than simple immediate hypersensitivity. Immune interaction between coexisting neoplasms could lead to an increased susceptibility to develop side effects to specific chemotherapy agents. Relative lymphocytosis constantly shown in case 1 may be representative of such a ‘hypersensitive state’.

In conclusion, lymphoproliferative disorders can be associated with solid tumors and complicate their disease course. The time of manifestation with respect to the primary cancer and the type of LPD will determine the optimal treatment type. Treatment can often favor management of the cancer, which has a poorer prognosis. Early detection and specific diagnosis programs should be therefore perceived for cancer survivors, life-long. In addition, careful attention should be paid to the choice of the chemotherapy agents used in association, due to the potentially increased risk of drug reactivity.

## Figures and Tables

**Table I:** Citofluorimetric analysis of lymphoid population in Case 1.

Antigene	Frequency (%)
CD3^+^	54
**CD5^+^**	**54**
CD3^+^ CD5^+^	54
CD3^−^ CD5^+^	0
**CD19^+^**	**34**
**CD20^+^**	**27**^a^
**CD19^+^** **CD5^+^**	**0.7**
CD19^+^ CD10^+^	0
CD19^+^ CD23^+^	2
**CD19^+^** **FMC7^+^**	**26**
**CD19^+^** **CD38^+^**	**NS**
**CD19^+^** **CD49d^+^**	**NS**
CD19^+^ CD20^+^	27
CD19^+^ CD103^+^	0
**CD19^+^** **CD11c^+^**	**5**
CD19^+^ sκ^+^	1
**CD19^+^** **sλ^+^**	**33**

Bold text denotes an expansion of B lymphocytes, CD19^+^ and CD20^+^ (normal value below 20%) and the presence of a clonal restriction for λ chains is also observed.

a High.

NS, not significant.
